# Mental Research and Government Aid

**Published:** 1913-05-31

**Authors:** 


### Mental Research and Government Aid.

MR. McKENNA'S STATEMENT AT CARDIFF.

THE Home Secretary, Mr. McKenna, as we note elsewhere, visited Cardiff Mental Hospital last week, and the object of his visit was explained by the Lord Mayor in proposing his health at a luncheon in the City Hall.

The Lord Mayor, Mr. Alderman Thomas, who is Chairman of the Mental Hospital Committee, in the course of his speech, said it was an open secret that their object in inviting Mr. McKenna to visit the Cardiff Mental Hospital was to induce him to make a new departure by instituting Government grants to those local authorities who were progressive enough to establish departments of research into the causation and cure of mental diseases. The treatment of insanity had not hitherto been dealt with so effectively in this country as on the Continent, and there was a prevailing idea here that when a person was taken to what was mis-termed a lunatic asylum—a term which he abhorred and strongly objected to—the patient had no hope of a cure. That was fast becoming an exploded idea. In this research work, so successfully carried on at Cardiff by Dr. Goodall and his staff of experts, no exception was ever taken by the City Council to expenditure that would enable their local experts to solve the problems more effectively. They wanted every similar institution in the country, with their own, to advance in real usefulness, but there were some authorities who were not in a position to spend money on research work as was done in Cardiff, and his Corporation now asked the Home Office to bring pressure to bear upon all the authorities concerned to help in the work of research in their own institutions, and to assist those authorities to do so by means of grants.

The Home Secretary, in his reply, first caused great enthusiasm by saying, "I am certain that to-day Cardiff is the capital of Wales," and then said that no institution in the City was of greater importance, in one sense, than the mental hospital. It had been a revelation to him to see the magnificent—he could use no other word —preparation which they had made in Cardiff for the care and cure of those who were mentally afflicted. Their institution had rightly been called a mental hospital. They received their patients with the intention of curing them, and he had learnt from Dr. Goodall—in the praise of whom he heartily wished to join—that last year they had been able to discharge cured no less than 60 per cent. of the patients admitted during that period. That was a remarkable achievement. The Cardiff Mental Hospital stood not merely second to none in Wales in the development they had made in that particular branch of science, but it stood second to none in the whole of the United Kingdom, and it stood to the glory of the name of Cardiff that their mental hospital could not fail to be regarded as the pioneer in the great department of medical curative work. He would like to be in a position to say straightaway that he would meet the wishes of the Lord Mayor and Corporation and make an announcement of future assistance from the Government, but, unfortunately, although the Home Office was directly concerned with the Lunacy Commissioners, still the last word in all questions of finance had to be said by the Treasury, and the utmost he could do at the moment was to assure them that he would express to the Chancellor of the Exchequer, in the most cordial terms of which he was capable, his appreciation of the admirable work which was being done at Cardiff, and of the real necessity for the extension of that work not merely at Cardiff, but at other great centres, like London, the West Riding, and Lancashire, where an attempt of a similar character was being made to effect a far larger proportion of cures than had been possible in the past.

